# Early identification of patients at risk of long-term critical illness-associated physical disability: is it possible?

**DOI:** 10.1186/s13054-014-0629-3

**Published:** 2014-11-19

**Authors:** Evelyn J Corner, Stephen J Brett

**Affiliations:** Chelsea and Westminster NHS Foundation Trust and Imperial College London, Chelsea and Westminster Hospital, 369 Fulham Road, London, SW10 9NH UK; Centre for Perioperative Medicine and Critical Care Research, Imperial College NHS Healthcare Trust and Imperial College London, Hammersmith Hospital, Du Cane Road, London, W12 0HS UK

## Abstract

ICU-acquired weakness can hinder and determine the course of recovery from critical illness, leading to life-changing disability. Risk factors include multiorgan failure and prolonged bed rest; however, no prognostic model or screening tool for new-onset disability has been established to date. With no way of targeting the at-risk population, it is difficult to demonstrate the benefit of rehabilitation interventions in research and prioritize resources clinically. In a recent issue of *Critical Care*, Schandl and colleagues aimed to establish a predictive screening tool for new-onset disability using 23 possible predictors. They found that using the following risk factors – low educational level, fractures, reduced core stability and length of ICU stay over 2 days – they were able to develop a risk score predictive of disability at 2 months after hospital discharge. These investigators propose that this will help to identify patients requiring follow-up and may increase the power to detect change in interventional studies. Whilst this is promising work, further validation is essential: firstly, to make it a clinically workable tool in terms of appropriate ‘cut offs’; secondly, to ensure that it is transferable in different socio-economic environments; and finally, to make sure that those identified as ‘at risk’ are those that would benefit the most from targeted intervention.

## Introduction

In a recent issue of *Critical Care*, Schandl and colleagues describe a study which had the aim of establishing a predictive tool for new-onset disability [1]. The authors propose that this will help to identify patients requiring follow-up and may increase the power to detect change in interventional studies.

The long-term sequelae of critical illness are well documented [2]. Significant muscle loss occurs at a rate of up to 15% within 1 week of multiorgan failure [3]. This, coupled with the negative effect of bed rest, can lead to life-changing disability. Over the past decade, mortality rates from severe sepsis have improved substantially (35% in 2001 to 18.4% in 2012) [4]; however, for patients this comes at a price. There has also been a significant increase in referrals for ongoing rehabilitation following hospital discharge [4,5]. Hence, the focus has shifted from reducing mortality to minimizing morbidity [6].

Early rehabilitation in the ICU is a safe, cheap and efficacious way of helping patients to achieve their optimal outcome [7]. However, although evidence is accumulating that early rehabilitation works, beyond those with obvious injury we have no way of identifying patients likely to develop physical disability, and hence to benefit from early rehabilitation [6]. This issue also creates difficulties for patient stratification in clinical trials.

Schandl and colleagues set out to address this issue by developing a method of predicting new-onset disability [1]. If a predictive model could be established, it would facilitate targeted intervention in research and clinical practice.

## Main text

Schandl and colleagues assessed the baseline level of disability in a general ICU cohort (*n* =232) using the Katz Activity of Daily Living Index. They reassessed disability using the ADL-staircase questionnaire and recorded whether the patient was off work for physical reasons 2 months after discharge [1]. Patients were then separated into two groups: physical disability, and no physical disability.

Twenty-three potential predictors of disability were collected for each participant.

Using multivariable logistic regression, the investigators developed a screening instrument using four predictors; ICU length of stay >48 hours, low educational level (elementary or lower), fractures, and reduced core stability (that is, unable to maintain independent sitting balance at ICU discharge). A risk score can then be plotted to give a percentage probability of new-onset disability.

This is the first time a predictive model for long-term disability has been developed and hence is important, novel work in this field. The heterogeneity of ICU patients makes researching rehabilitation strategies to improve function very difficult. If this model can be used to identify the at-risk patients, it would enable targeted interventional studies into rehabilitation strategies. The model could also inform patients about their recovery trajectory, and may help in the design of future rehabilitation services. However, as acknowledged by the authors, this model needs thorough prospective investigation before widespread adoption. Its sensitivity in other populations needs evaluation in studies using meaningful functional descriptors, which reflect disability adequately and are responsive to change in this cohort [5,8].

The constituent components of this screening model warrant discussion. The presence of fractures as a predictor of disability is unsurprising given fracture-healing time and restrictions placed by orthopedic services. Certainly for some, early intervention in this group would be beneficial. However, as rehabilitation prior to removal of restrictions can be limited, and these patients may see sudden improvements once restrictions are removed, the presence of fractures may falsely identify a specific group of patients whom may not benefit the most from early intervention.

If a patient is in the ICU for over 48 hours, this model gives them roughly a 33% probability of developing new-onset disability – this is the lowest predictor of risk. If the patient has reduced core stability or fractures, they have a roughly 50% chance of disability – the cutoff point when using this model for research and clinical practice therefore needs consideration. If the lowest risk stratification is applied and patients are recruited into studies after 2 days, the issue of heterogeneity would persist. With more stringent criteria, however, a significant proportion of patients requiring rehabilitation may be missed.

The core stability component is dichotomous, segregating patients at a low level of function. This has face validity for identifying a very disabled cohort; however, severity of disability does not always correlate with quality of life. Furthermore, the presence of disability does not guarantee rehabilitation potential; that is, the ability to improve. Hence, higher functioning patients may actually be the ones that benefit the most from targeted intervention, a question not yet addressed in the literature.

Lower educational level was also predictive. The transferability of this component needs thorough evaluation of different socioeconomic cultures and educational systems. The authors suggest that patients with a higher educational level have better problem-solving and goal-setting strategies. This is a potentially important, although sensitive, concept and provides a cogent argument for an interventional study into rehabilitation goal setting in ICU patients.

Methodological considerations from this study include the measures used to define disability; the responsiveness of the two tools is not addressed, neither tool is validated in the ICU, and the pre- and post-disability measures were different.

## Conclusions

This work shows exciting progress in the identification of new-onset disability following critical illness. The study provides a simple, objective model to identify an at-risk patient group for targeted intervention. However, as acknowledged by the authors, external validation is required with different populations and settings. A randomized controlled trial to investigate the ability of this model to screen in appropriate patients for targeted rehabilitation may be a subsequent natural step, and would provide opportunity to explore any concerns regarding its component parts. Such work is vital to ensure that survivors of critical illness are survivors, and not victims.
